# Prevalence and Co-Occurrence of Substance Use Disorders and Independent Mood and Anxiety Disorders: Results From the National Epidemiologic Survey on Alcohol and Related Conditions

**Published:** 2006

**Authors:** Bridget F. Grant, Frederick S. Stinson, Deborah A. Dawson, S. Patricia Chou, Mary C. Dufour, Wilson Compton, Roger P. Pickering, Kenneth Kaplan

**Affiliations:** From the Department of Health and Human Services, National Institutes of Health, National Institute on Alcohol Abuse and Alcoholism (NIAAA) Laboratory of Epidemiology and Biometry, Division of Intramural Clinical and Biological Research, Bethesda, MD: Drs. Grant, Stinson, Dawson, and Chou and Mr. Pickering. From the Office of the Director, NIAAA: Dr. Dufour. From the Division of Epidemiology, Services, and Prevention Research, of the National Institute on Drug Abuse, Bethesda, MD: Dr. Compton. From the Demographic Surveys Division, U.S. Census Bureau, Suitland, MD: Mr. Kaplan

## Abstract

**Background:**

Uncertainties exist about the prevalence and comorbidity of substance use disorders and independent mood and anxiety disorders.

**Objective:**

To present nationally representative data on the prevalence and comorbidity of Diagnostic and Statistical Manual of Mental Disorders, Fourth Edition (DSM–IV) alcohol and drug use disorders and independent mood and anxiety disorders (including only those that are not substance induced and that are not due to a general medical condition).

**Design:**

Face-to-face survey.

**Setting:**

The United States.

**Participants:**

Household and group quarters residents.

**Main Outcome Measures:**

Prevalence and associations of substance use disorders and independent mood and anxiety disorders.

**Results:**

The prevalences of 12-month DSM–IV independent mood and anxiety disorders in the U.S. population were 9.21 percent (95 percent confidence interval [CI], 8.78 percent–9.64 percent) and 11.08 percent (95 percent CI, 10.43 percent–11.73 percent), respectively. The rate of substance use disorders was 9.35 percent (95 percent CI, 8.86 percent–9.84 percent). Only a few individuals with mood or anxiety disorders were classified as having only substance-induced disorders. Associations between most substance use disorders and independent mood and anxiety disorders were positive and significant (p < .05).

**Conclusions:**

Substance use disorders and mood and anxiety disorders that develop independently of intoxication and withdrawal are among the most prevalent psychiatric disorders in the United States. Associations between most substance use disorders and independent mood and anxiety disorders were overwhelmingly positive and significant, suggesting that treatment for a comorbid mood or anxiety disorder should not be withheld from individuals with substance use disorders.

Substance use disorders and mood and anxiety disorders are widespread among the general population^1^–^3^ and are associated with substantial societal and personal costs.^4^–^7^ Furthermore, national epidemiologic surveys^1^–^3^ and numerous clinical studies^8^–^12^ consistently indicate that substance use disorders and mood and anxiety disorders have strong associations when considered on a lifetime basis. However, consensus has not been achieved on the meaning and implications of the lifetime association of these widespread disorders. Recent work in the general population separating past and current disorders has clarified that intoxication or withdrawal effects do not entirely account for the association,^13^ as had been asserted earlier.^14^–^17^ However, the nature of current or recent co-occurrence of substance and mood or anxiety disorder remains largely unexamined and poorly understood. Relative to lifetime disorders, current co-occurrence has much more salience in its public health and clinical implications. Thus, an important gap in knowledge about comorbidity remains.

One factor that has persistently hindered a better understanding of the relationship between substance use disorders and mood and anxiety disorders is diagnosis. The diagnosis of current mood or anxiety disorders among active substance abusers is complicated by the fact that many symptoms of intoxication and withdrawal from alcohol and other substances resemble the symptoms of mood and anxiety disorders. The diagnostic challenge among individuals with current substance use disorders has been to devise diagnostic criteria and measurement techniques that differentiate between intoxication and withdrawal symptoms and the symptoms of psychiatric disorders. This distinction is potentially crucial for etiologic research and treatment studies.

The DSM–IV^18^ represented a major departure from previous nomenclature in the importance placed on the independent and substance-induced distinction and the clarity and specificity of the guidelines for making the distinction. Among individuals with substance use disorders, independent DSM–IV diagnoses of mood or anxiety disorders can be made two ways. First, the full mood or anxiety syndrome is established before substance use. Second, the mood or anxiety syndrome persists for more than 4 weeks after the cessation of intoxication or withdrawal. In contrast, substance-induced disorders are defined as those occurring only during periods of substance use (or remitting shortly thereafter). These specific diagnostic criteria provide a clearly defined situation for studying the association of substance use disorders and mood and anxiety disorders that eliminates potential diagnostic confusion arising from misdiagnosis of intoxication or withdrawal effects.

There have been recent attempts to respond to the challenge of differentiating independent and substance-induced mood and anxiety disorders in clinical samples, focusing on patients with substance use disorders.^14^–^17^ These differentiations were based on the occurrence of substance use disorders rather than on substance use per se. In these studies, independent mood or anxiety disorders were defined as episodes occurring either before the lifetime initial onset of a substance use disorder or during a period of remission lasting at least 3 months. Remission was defined as abstinence. Other episodes of mood or anxiety disorders were classified as substance-induced disorders. The distinction between independent and substance-induced disorders in these studies is problematic in several ways. First, retrospective reports of chronological sequences occurring many years earlier may be inaccurate. Second, basing the distinction on substance use disorders rather than on periods of substance use leaves open the possibility that independent psychiatric disorders occurring during periods of nondiagnosable substance use were missed. Third, the clinical assessment methods in these studies did not ascertain episodes of independent mood and anxiety disorders beginning during periods of drinking or drug use and persisting longer than 1 month after the cessation of use (as specified in DSM–IV), thus potentially missing further independent cases. From an epidemiologic perspective, however, the most serious problem with research on comorbidity in treated samples is that the samples of subjects do not represent the underlying populations. Avoiding this problem requires epidemiologic methods.

To our knowledge, no epidemiologic survey has used the DSM–IV definitions of independent and substance-induced disorders to investigate comorbidity between substance use disorders and mood and anxiety disorders. The Epidemiologic Catchment Area survey, conducted in the early 1980s, based its diagnoses on the *Diagnostic and Statistical Manual of Mental Disorders, Third Edition* (DSM–III), 20 which had little relevance to today’s diagnostic concepts, in either the criteria for substance use disorders or the characterization of the independent and substance-induced distinction. The 1990–1992 National Comorbidity Survey (NCS)^2^ used *Diagnostic and Statistical Manual of Mental Disorders, Third Edition, Revised* (DSM–III–R)^21^ criteria. While the DSM–III–R definitions of substance use disorders were more similar to those in the DSM–IV, the handling of substance use disorders was quite different. The more recent 2001–2002 NCS–2 and NCS–Replication were intended to yield DSM–IV diagnoses. However, the NCS–2 and NCS–Replication assessment instruments did not differentiate between independent and substance-induced disorders but, rather, asked respondents if they thought their mood or anxiety disorder was due to drinking or drug use or to a physical illness. Clearly, such opinions may differ from the intent and the specific definitions provided in the DSM–IV.

In addition, measurement of substance use disorders itself has hindered examination of the independent and substance-induced distinction and its effect on the comorbidity between substance use disorders and mood and anxiety disorders in the general population. In the Epidemiologic Catchment Area survey^23^ and the NCS–2,^2^ substance dependence was not measured as a syndrome, because clustering in time of the required number of symptoms was not assessed. In addition, the NCS–2 and NCS–Replication do not yield drug-specific diagnoses, but rather produce polysubstance dependence diagnoses for which dependence criteria are met for substances as a group, but not necessarily for any specific drug. In addition, the symptoms of abuse are used as screeners for dependence, with negative responses to abuse questions leading to a skip past questions on dependence. This leads to an undercount of about one-third of the cases of dependence in the general population.^24^ However, more seriously, it leads to a loss of specific types of cases, because women with dependence are much less likely to have symptoms of abuse than men.^24^ Women are also the individuals most likely to have mood and anxiety disorders, so missing these cases of dependence without abuse symptoms is likely to lead to underestimates of prevalence and comorbidity.

Because of the widespread prevalence of mood, anxiety, and substance use disorders and their associated disabilities and social costs, an accurate understanding of their comorbidity is crucial to prevention and treatment. This report presents data from a major national survey designed to overcome the problems of previous epidemiologic surveys on comorbidity. This survey, the National Institute on Alcohol Abuse and Alcoholism’s National Epidemiologic Survey on Alcohol and Related Conditions (NESARC),^25^,^26^ covers the comorbidity of DSM–IV substance use disorders and nine independent mood and anxiety disorders in a nationally representative U.S. sample of 43,093 respondents. To our knowledge, this is the largest comorbidity survey ever conducted. The sample size allows for accurate estimation of current comorbidity and/or rare conditions. More important, to our knowledge, NESARC is the first and only national survey to use the specific DSM–IV definitions of independent and substance-induced disorders to determine if mood, anxiety, and substance use disorders are associated even when substance-induced disorders are ruled out. Furthermore, NESARC operationalized alcohol and drug dependence as syndromes, measured drugspecific diagnoses of dependence, and ascertained alcohol and drug dependence among all alcohol and drug users, regardless of whether they had an abuse diagnosis. The study also provides comorbidity rates separately for respondents seeking treatment for alcohol, drug, and emotional problems, because rates and patterns of comorbidity associated with the presenting complaint are most germane to practicing clinicians.

## Methods

### Sample

Wave I of NESARC is a nationally representative face-to-face survey of 43,093 respondents, 18 years and older, conducted by the National Institute on Alcohol Abuse and Alcoholism in 2001–2002,^25^,^26^ The target population of NESARC is the civilian noninstitutionalized population residing in the United States, including Alaska and Hawaii. The housing unit sampling frame of NESARC was the U.S. Bureau of the Census Supplementary Survey.^25^ NESARC also included a group quarters sampling frame derived from the Census 2000 Group Quarters Inventory.^25^ The group quarters sampling frame captures important subgroups of the population with heavy substance use patterns not often included in general population surveys. These included the military living off base, boarding houses, rooming houses, nontransient hotels and motels, shelters, facilities for housing workers, college quarters, and group homes. Hospitals, jails, and prisons were not among the group quarters sampled. The overall survey response rate was 81.0 percent, substantially higher than that of other surveys of this kind.

Black and Hispanic households were oversampled. The oversampling procedure increased the percentage of non-Hispanic Black households in the sample from 12.3 percent to 19.1 percent (*n* = 8,245) and the percentage of Hispanic households from 12.5 percent to 19.3 percent (*n* = 8,308). Black and Hispanic persons were oversampled because these subgroups have been underrepresented in previous comorbidity surveys. One sample person from each household or group quarters unit was randomly selected for interview, and young adults, ages 18 to 24, were oversampled at a rate of 2.25 times that of other members in the household.

The NESARC sample was weighted to adjust for the probabilities of selection of a sample housing unit or housing unit equivalent from the group quarters sampling frame, nonresponse at the household and person levels, the selection of one person per household, and oversampling of young adults. Once weighted, the data were adjusted to be representative of the U.S. population for various sociodemographic variables, including region, age, sex, race, and ethnicity, based on the 2000 Decennial Census. The sociodemographic distribution of the NESARC sample is shown in Table 1.

### Substance Use Disorder Assessment

The diagnostic interview used to generate the diagnoses presented in this report is the National Institute on Alcohol Abuse and Alcoholism’s Alcohol Use Disorder and Associated Disabilities Interview Schedule—DSM–IV Version (AUDADIS–IV),^27^ a state-of-the-art structured diagnostic interview designed for use by lay interviewers. The DSM–IV diagnoses reported herein, and included in the AUDADIS–IV, were alcohol- and drug-specific abuse and dependence (excluding nicotine dependence), major depression, dysthymia, mania, hypomania, panic disorder with and without agoraphobia, social phobia, specific phobia, and generalized anxiety disorder. Not all mood and anxiety disorders were assessed in Wave 1 of NESARC because of time and space constraints. However, Wave 2 of NESARC will assess post-traumatic stress disorder.

The AUDADIS–IV included an extensive list of symptom questions that separately operationalized DSM–IV for substance use disorders, including alcohol abuse and dependence and drug-specific abuse and dependence for eight classes of drugs, including sedatives, tranquilizers, opiates (other than heroin or methadone), stimulants, hallucinogens, cannabis, cocaine (including crack cocaine), and inhalants/solvents. Consistent with the DSM–IV, 12-month (current) AUDADIS–IV diagnoses of alcohol abuse required a respondent to meet at least 1 of the 4 criteria defined for abuse in the 12-month period preceding the interview. The AUDADIS–IV dependence diagnoses required the respondent to satisfy at least 3 of the 7 DSM–IV criteria for dependence during the past year. The drug-specific diagnoses of abuse and dependence were derived using the same algorithm previously described for alcohol use disorders.

The test–retest reliabilities of AUDADIS–IV alcohol and drug disorder measures were excellent, exceeding *kappa* = 0.74 for alcohol diagnoses and *kappa* = 0.79 for drug diagnoses.^28^–^32^ The discriminant and convergent,^33^,^34^ concurrent,^45^,^46^ construct,^47^–^49^ and population^50^ validity of the AUDADIS–IV alcohol and drug use disorder diagnoses also has been well documented, including in the World Health Organization/National Institutes of Health Reliability and Validity Study.^41^,^44^,^46^,^51^–^53^

### Mood and Anxiety Disorder Assessment

Independent and substance-induced disorders were defined for respondents who met the criteria for specific mood and anxiety disorders occurring during the past 12 months. Disorders were classified as independent if (1) the respondent abstained from alcohol and drug use in the past 12 months; (2) the episode(s) did not occur in the context of alcohol or drug intoxication or withdrawal; (3) the episode(s) occurred before alcohol or drug intoxication or withdrawal; or (4) the episode(s) began after alcohol or drug intoxication or withdrawal, but persisted for more than 1 month after the cessation of alcohol or drug intoxication or withdrawal. Substance-induced disorders were defined as episodes that began after alcohol and/or drug intoxication and/or withdrawal, but either (1) were not associated with a period of at least 1 month of abstinence or (2) did not persist for more than 1 month after the cessation of alcohol or drug intoxication or withdrawal.

Respondents were classified with a 12-month independent mood or anxiety disorder if none or only some of their episodes were substance induced. Respondents were classified with a substance-induced disorder if all of their episodes in the past 12 months were substance induced.

The prevalence of major depression reported herein ruled out bereavement. All mood and anxiety disorders due to general medical conditions also were ruled out. The latter were defined as those occurring during the past 12 months when the respondent was physically ill or recovering from being physically ill, with the additional requirement that a physician or other health professional confirmed that the episode was related to the respondent’s physical illness or medical condition. This definition also required the onset of the mood or anxiety disorder to begin during the time of a physical illness or during recovery from it.

The test–retest reliabilities of AUDADIS–IV measures of DSM–IV mood and anxiety disorders were fair to good, ranging from *kappa* = 0.42 for specific phobia to *kappa* = 0.64 for major depression.^28^,^30^ The validity of current mood and anxiety disorders was assessed in a series of linear regression analyses, using the NESARC data, that examined the associations between each mood and anxiety disorder and Short-Form-12v2^54^ mental disability scores, controlling for age, personality disorders, current comorbid alcohol and drug use disorders, and all other comorbid mood and anxiety disorders. The Short-Form-12v2 is a reliable and valid measure of generic quality of life used in large population surveys. In the present analyses, the focus was on four Short-Form-12v2 mental disability scores (the mental component summary score, the social functioning score, the role emotional function score, and the mental health score), reflecting general mental health functioning. With the exception of hypomania, all mood and anxiety disorders assessed in NESARC were highly significant (*p* < .003 – *p* < .001) predictors of the mental component summary, social functioning, role emotional, and mental health scores. Respondents with these current mood and anxiety disorders had significantly greater disability and social/occupational dysfunction than respondents who did not have the particular mood or anxiety disorder. A diagnosis of hypomania was a significant predictor (*p* = .049) of the social functioning score.

### Twelve-Month Treatment Use

The NESARC respondents were asked about 12-month treatment use separately for alcohol, drugs, and each specific mood or anxiety disorder. Alcohol treatment use was defined as seeking help for alcohol problems in the 12 months preceding the survey, at any of the following agencies or from any of the following health professionals: human services, including family services or other social service agencies; emergency departments or crisis centers; alcohol specialty services, including alcohol or drug detoxification wards or clinics, outpatient clinics, outreach programs, or day or partial patient programs; inpatient wards of a psychiatric or general hospital or community mental health facilities; alcohol or drug rehabilitation programs; halfway houses; and visits to a physician, psychiatrist, psychologist, social worker, or other health professional. The 12-month drug treatment use questions paralleled those of the alcohol treatment use questions, with the exception that methadone maintenance programs were also included as drug specialty services.

Twelve-month treatment use was ascertained separately for each specific mood and anxiety disorder. Respondents were classified as receiving treatment in the past 12 months if they: (1) visited a counselor, therapist, physician, psychologist, or person like that to get help for a mental disorder; (2) were a patient in a hospital for at least 1 night related to a mental disorder; (3) visited an emergency department to get help for a mental disorder; or (4) were prescribed medications for a mental disorder.

### Interviewer Training and Field Quality Control

Approximately 1,800 experienced lay interviewers from the U.S. Census Bureau administered NESARC using laptop computer–assisted software that included built-in skip, logic, and consistency checks. On average, the interviewers had 5 years of experience working on Census and other health-related national surveys. The interviewers completed 10 days of extensive training. This was standardized through centralized training sessions under the direction of the National Institute on Alcohol Abuse and Alcoholism and Census headquarters staff.

Regional supervisors recontacted a random 10 percent of all respondents for quality control purposes and for verification of the accuracy of the interviewers’ performance. In addition, 2,657 respondents were randomly selected to participate in a reinterview study after completion of their NESARC interview. These interviews not only served as an additional check on survey data quality but formed the basis of a test–retest reliability study^30^ of AUDADIS–IV modules introduced in NESARC.

### Statistical Analysis

Cross-tabulations were used to calculate prevalences, comorbidity, and 12-month treatment use for alcohol, drug, mood, and anxiety disorders. Odds ratios (ORs) were used to study associations between substance use disorders and independent mood and anxiety disorders. Standard errors and 95 percent confidence intervals were estimated using a software package (SUDAAN^55^) that uses Taylor series linearization to adjust for the design effects of complex sample surveys like NESARC.

## Results

### Prevalence of Mood and Anxiety Disorders

The 12-month prevalences of independent mood and anxiety disorders were 9.21 percent and 11.08 percent in the total sample, respectively (Table 2). The prevalences of substance-induced mood and anxiety disorders among respondents with any mood or anxiety disorder in the total sample and among respondents with and without a current substance use disorder were small, less than 1.0 percent. Of the approximately 19.3 million adults who had a current mood disorder, only 202,211 experienced episodes that were classified exclusively as substance induced. Similarly, among those with a current anxiety disorder (23.0 million), only a few (50,980) experienced episodes that were exclusively classified as substance induced. Of those respondents who were classified as having at least one current independent mood or anxiety disorder, only 7.35 percent and 2.95 percent, respectively, reported experiencing independent and substance-induced episodes during the year preceding the survey.

### Prevalence of Substance Use Disorders

The 12-month prevalences of any substance, any alcohol, and any drug use disorders were 9.35 percent, 8.46 percent, and 2.00 percent, respectively (Table 3). The rate of cannabis use disorder was 1.45 percent, far exceeding the rates of other drug-specific use disorders (0.02 percent for inhalant/solvent abuse to 0.35 percent for opioid use disorders). The rates for abuse exceeded those for dependence regardless of the specific substance use disorder examined.

### Co-Occurrence of Substance Use Disorders and Mood and Anxiety Disorders

The 12-month associations between substance use disorders and independent mood and anxiety disorders are shown in Table 4 in the form of ORs. The overall pattern of ORs is overwhelmingly positive, with 84.8 percent of the disorder-specific ORs positive (i.e., >1.0) and statistically significant. All independent mood and anxiety disorders were strongly and consistently related to alcohol and drug use disorders (ORs, 1.3–13.9). Any drug abuse also was significantly related to all independent mood and anxiety disorders (ORs, 1.6–4.2). The exception to the overall pattern was the level of association between alcohol abuse and specific independent mood and anxiety disorders, which was not always significant. All the independent mood and anxiety disorders were consistently more strongly related to alcohol and drug dependence than to drug abuse. Mania was more strongly related to the substance use disorders (ORs, 1.4–13.9) than any other mood or anxiety disorder. Among the anxiety disorders, panic disorder with agoraphobia was most strongly associated with substance use disorders (ORs, 1.9–10.5).

As indicated by the entry in the upper left corner of Table 5, 19.67 percent of the respondents with any substance use disorder had at least one independent mood disorder during the same 12-month period. Furthermore, 17.71 percent had at least one independent anxiety disorder. Among respondents with any substance use disorder, 3.30 percent to 14.50 percent also had a specific mood disorder, and 1.46 percent to 10.54 percent had a specific anxiety disorder. These rates were consistently lower for abuse than for dependence and highest for any drug dependence. Respondents with substance use disorders were more likely to have major depression and specific phobia than any other mood or anxiety disorder.

### Prevalence of Substance Use Disorders Among Respondents With Mood or Anxiety Disorders

Among respondents with any 12-month mood disorder, 19.97 percent had at least one substance use disorder, and among those with any 12-month anxiety disorder, 14.96 percent had at least one substance use disorder (Table 6). Among respondents with specific mood disorders, 18.07 percent to 27.91 percent also had at least one substance use disorder. This was also true of 13.83 percent to 24.15 percent of the respondents with specific anxiety disorders. Prevalences were consistently lower for abuse than for dependence. Respondents with panic disorder with agoraphobia and generalized anxiety disorder were more likely than those with other mood and anxiety disorders to have a substance use disorder.

### Prevalence of Substance Use Disorders Among Respondents With Mood and Anxiety Disorders Who Sought Treatment

The percentage of respondents with at least one 12-month independent mood disorder who sought treatment in the past 12 months was 25.81 percent, while the corresponding percentage for respondents with at least one independent anxiety disorder was 12.13 percent (Table 7). Treatment use was greater for those with dysthymia, major depression, and mania than for those with hypomania. Among respondents with anxiety disorders, treatment use was greater for those with panic disorder, with and without agoraphobia, and generalized anxiety disorder than for those with social and specific phobias.

Among respondents reporting specific independent mood disorders, between 18.54 percent and 30.97 percent had a comorbid substance use disorder, primarily an alcohol use disorder. Among respondents reporting specific independent anxiety disorders who sought treatment, 15.38 percent to 21.89 percent had a comorbid substance use disorder, again primarily an alcohol use disorder.

### Prevalence of Mood and Anxiety Disorders Among Respondents With Substance Use Disorders Who Sought Treatment

Only 5.81 percent and 13.10 percent of respondents who had a 12-month alcohol use disorder or a 12-month drug use disorder, respectively, sought treatment for their particular substance use disorder during that same period (Table 8). Among those who sought treatment for an alcohol use disorder, 40.69 percent, 33.38 percent, and 33.05 percent had at least one independent mood disorder, independent anxiety disorder, or drug use disorder, respectively. Among respondents with any drug use disorder who sought treatment for that disorder, 60.31 percent had at least one independent mood disorder, 42.63 percent had at least one independent anxiety disorder, and 55.16 percent had a comorbid alcohol use disorder.

## Comments

The major findings of this study document the extremely high rates of substance use disorders and independent mood and anxiety disorders in the U.S. population and confirm the strength of associations between them. The prevalence of any current independent mood disorder was 9.21 percent, representing 19.2 million adult Americans. The prevalence of any current independent anxiety disorder was slightly higher, 11.08 percent, representing 23 million U.S. adults. The rate of any current substance use disorder was only slightly greater than that estimated for independent mood disorders, 9.35 percent, representing 19.4 million U.S. adults. Almost 9 percent (17.6 million adult Americans) had an alcohol use disorder, while 2 percent (4.2 million adult Americans) had a drug use disorder. Furthermore, about 20 percent of all persons in the general population with a current substance use disorder had at least one current independent mood disorder, and 18 percent had at least one current independent anxiety disorder. Similarly, about 20 percent of the individuals with at least one current independent mood disorder had a comorbid substance use disorder, while about 15 percent of the individuals with at least one 12-month independent anxiety disorder had a substance use disorder. More important, this study also demonstrated that a few individuals in the general population experienced current mood (202,211 adult Americans) or anxiety (50,980 adult Americans) disorders that were only substance induced.

Of considerable clinical relevance is the finding that 40.7 percent of the individuals with a current alcohol use disorder who sought treatment during the same period had at least one current independent mood disorder, while more than 33 percent had at least one current independent anxiety disorder. Among individuals with a current drug use disorder who sought treatment, about 60 percent and 43 percent had at least one current independent mood or anxiety disorder, respectively. Similarly, among individuals with at least one current independent mood or anxiety disorder who sought treatment, about 20 percent and 16 percent, respectively, had a current substance use disorder that was more likely to be an alcohol than a drug use disorder. This suggests that the predominance of substance-induced (approximately 60 percent) rather than independent mood or anxiety disorders found in several recent clinical studies^15^–^17^ of substance abusers was most likely due to diagnostic methods that do not entirely conform to the DSM–IV guidelines for differentiating independent from substance-induced disorders. Regardless of the relative prevalence of independent and substance-induced disorders, however, substance-induced mood or anxiety disorders among individuals with substance use disorders are serious conditions. For example, when diagnosed carefully according to DSM–IV guidelines, substance-induced disorders have been shown to increase the risk for poor outcome of substance dependence^56^ and lifetime number of suicide attempts.^57^ Additional longitudinal research is needed to examine differences in the course and prognosis of chronic substance-induced disorders and independent mood and anxiety disorders in treated samples.

Taken together, the NESARC results provide clear and persuasive evidence that mood and anxiety disorders must be addressed by alcohol and drug treatment specialists and that substance use disorders must be addressed by primary care physicians and mental health treatment specialists. These results highlight the need for all individuals in treatment to be fully assessed for the presence or absence of a range of psychiatric disorders. Furthermore, the results underscore the importance of past and ongoing development of improved treatments for those individuals meeting the criteria for two or more disorders.^58^–^62^ Moreover, these results strongly suggest that treatment for a mood or anxiety disorder should not be withheld from those with substance use disorders in stable remission on the assumption that most of these disorders are due to intoxication or withdrawal. Left untreated, such mood disorders have been shown to lead to relapse of substance dependence^56^ and can also be fatal, as many former substance abusers with severe untreated independent depression will die by suicide. Short of this ultimately adverse outcome, independent mood and anxiety disorders, particularly among individuals who have a comorbid substance use disorder, are immensely disabling.^4^–^7^

From an etiologic perspective, this study does not resolve questions regarding the casual mechanisms underlying the relationship between DSM–IV substance use disorders and independent mood and anxiety disorders. Prospective surveys have great potential to inform us about processes associated with comorbidity and will provide the vehicles for examining the sequencing of comorbid disorder onset. NESARC was designed with this paradigm in mind, and its second wave will be fielded in 2004–2005.

## Figures and Tables

**Table 1:** Characteristics of NESARC Respondents

Characteristics	Respondents		

	%	(S.E.)*	Total No.†
Sex			
Male	47.92	(0.31)	18518
Female	52.08	(0.31)	24575
Age			
18–29	21.80	(0.37)	8688
30–44	30.89	(0.32)	13382
45–64	31.06	(0.30)	12840
≥65	18.25	(0.33)	8205
Race or ethnicity			
White	70.89	(1.59)	24507
Black	11.07	(0.64)	8245
Native American	2.12	(0.16)	701
Asian or Pacific Islander	4.36	(0.53)	1332
Hispanic	11.56	(1.23)	8308
Personal income ($)			
0–19,999	47.25	(0.58)	21075
20,000–34,999	22.65	(0.36)	9999
35,000–69,999	21.96	(0.38)	9031
≥70,000	8.14	(0.38)	2988
Marital status			
Married or living with someone as if married	61.62	(0.47)	22081
Separated, divorced, widowed, or never married	38.38	(0.47)	21012
Education (years)			
0–11	15.65	(0.49)	7849
12	29.33	(0.55)	12547
13–15	30.14	(0.42)	12663
≥16	24.88	(0.62)	10034
Region			
Northeast	19.67	(3.41)	8209
Midwest	23.15	(3.18)	8991
South	35.21	(3.25)	16156
West	21.97	(3.51)	9737
Central city status			
Central city in MSA	29.53	(2.18)	15002
Not a central city in MSA	50.75	(2.14)	20295
Not in MSA	19.72	(1.61)	7796
Total	100.00		43093

Abbreviations: MSA = metropolitan statistical area; NESARC = National Epidemiologic Survey on Alcohol and Related Conditions.

* Based on weighted data.

† Based on unweighted data.

**Table 2:** Twelve-Month Prevalence of DSM–IV Mood and Anxiety Disorders With and Without Substance-Induced Disorders

Disorder	Respondents, % (S.E.)	

	Including Substance-Induced Disorders	Excluding Substance-Induced Disorders
**Among All Respondents**		
Any mood disorder	9.31 (0.22)	9.21 (0.22)
Major depression	7.17 (0.20)	7.06 (0.20)
Dysthymia	1.85 (0.09)	1.83 (0.09)
Mania	1.68 (0.08)	1.66 (0.08)
Hypomania	1.17 (0.07)	1.16 (0.07)
Any anxiety disorder	11.10 (0.33)	11.08 (0.33)
Panic disorder		
With agoraphobia	0.56 (0.05)	0.56 (0.05)
Without agoraphobia	1.58 (0.07)	1.55 (0.07)
Social phobia	2.76 (0.13)	2.75 (0.13)
Specific phobia	7.14 (0.26)	7.13 (0.26)
Generalized anxiety disorder	2.07 (0.10)	2.06 (0.10)

**Among Respondents With a 12-Month Substance Use Disorder**		
Any mood disorder	20.13 (0.80)	19.67 (0.78)
Major depression	15.15 (0.70)	14.50 (0.68)
Dysthymia	3.65 (0.36)	3.54 (0.36)
Mania	4.96 (0.41)	4.94 (0.41)
Hypomania	3.41 (0.33)	8.30 (0.33)
Any anxiety disorder	17.75 (0.81)	17.71 (0.81)
Panic disorder		
With agoraphobia	1.47 (0.26)	1.46 (0.26)
Without agoraphobia	2.90 (0.29)	2.86 (0.27)
Social phobia	4.72 (0.46)	4.72 (0.46)
Specific phobia	10.54 (0.67)	10.54 (0.67)
Generalized anxiety disorder	4.20 (0.41)	4.20 (0.41)

**Among Respondents Without a 12-Month Substance Use Disorder**		
Any mood disorder	8.19 (0.21)	8.13 (0.21)
Major depression	6.35 (0.20)	6.30 (0.19)
Dysthymia	1.67 (0.09)	1.66 (0.09)
Mania	1.34 (0.08)	1.32 (0.08)
Hypomania	0.94 (0.06)	0.94 (0.08)
Any anxiety disorder	10.42 (0.32)	10.39 (0.32)
Panic disorder		
With agoraphobia	0.47 (0.04)	0.47 (0.04)
Without agoraphobia	1.44 (0.08)	1.41 (0.08)
Social phobia	2.55 (0.13)	2.55 (0.13)
Specific phobia	6.79 (0.26)	8.78 (0.26)
Generalized anxiety disorder	1.85 (0.10)	1.84 (0.10)

**Table 3:** Twelve-Month Prevalence of DSM–IV Substance Use Disorders

Disorder	Respondents, % (S.E.)
Any substance use disorder	9.35 (0.25)
Any substance abuse	5.28 (0.19)
Any substance dependence	4.07 (0.14)
Any alcohol use disorder	8.46 (0.24)
Alcohol abuse	4.65 (0.18)
Alcohol dependence	3.81 (0.14)
Any drug use disorder	2.00 (0.10)
Any drug abuse	1.37 (0.08)
Any drug dependence	0.63 (0.05)
Sedative use disorder	0.16 (0.02)
Sedative abuse	0.09 (0.02)
Sedative dependence	0.07 (0.01)
Tranquilizer use disorder	0.13 (0.02)
Tranquilizer abuse	0.08 (0.02)
Tranquilizer dependence	0.05 (0.01)
Opioid use disorder	0.35 (0.05)
Opioid abuse	0.24 (0.04)
Opioid dependence	0.11 (0.02)
Amphetamine use disorder	0.16 (0.03)
Amphetamine abuse	0.09 (0.02)
Amphetamine dependence	0.07 (0.02)
Hallucinogen use disorder	0.14 (0.02)
Hallucinogen abuse	0.12 (0.02)
Hallucinogen dependence	0.02 (0.01)
Cannabis use disorder	1.45 (0.08)
Cannabis abuse	1.13 (0.06)
Cannabis dependence	0.32 (0.04)
Cocaine use disorder	0.27 (0.03)
Cocaine abuse	0.13 (0.02)
Cocaine dependence	0.13 (0.02)
Solvent/inhalant abuse*	0.02 (0.01)

* The base rate of solvent/inhalant dependence was virtually 0.0% in the sample.

**Table 4:** Twelve-Month Odds of DSM–IV Substance Use Disorders and Independent Mood and Anxiety Disorders*

Comorbid Disorder	Any Substance Use Disorder	Any Substance Abuse	Any Substance Dependence	Any Alcohol Use Disorder	Alcohol Abuse	Alcohol Dependence	Any Drug Use Disorder	Any Drug Abuse	Any Drug Dependence
Any mood disorder	2.8 (2.5–3.1)	1.4 (1.2–1.7)	4.5 (3.9–5.3)	2.6 (2.3–2.9)	1.3 (1.1–1.6)	4.1 (3.5–4.8)	4.9 (4.0–5.9)	2.7 (2.1–3.5)	12.5 (8.8–17.7)
Major depression	2.5 (2.2–2.9)	1.3 (1.1–1.6)	4.1 (3.4–4.8)	2.3 (2.0–2.6)	1.2 (1.0–1.5)	3.7 (3.1–4.4)	4.2 (3.4–5.2)	2.5 (1.9–3.3)	9.0 (6.5–12.7)
Dysthymia	2.2 (1.7–2.7)	1.1 (0.8–1.7)	3.4 (2.5–4.5)	1.7 (1.3–2.2)	0.8 (0.5–1.3)	2.8 (2.0–3.8)	5.3 (3.8–7.3)	2.6 (1.6–4.3)	11.3 (7.5–17.2)
Mania	3.9 (3.1–4.8)	1.5 (1.1–2.2)	8.4 (5.1–8.2)	3.5 (2.8–4.4)	1.4 (0.9–2.0)	5.7 (4.4–7.4)	7.4 (5.4–10.1)	4.2 (2.8–6.2)	13.9 (8.9–21.7)
Hypomania	3.6 (2.8–4.6)	1.9 (1.2–2.8)	5.1 (4.0–6.7)	3.5 (2.7–4.5)	1.7 (1.1–2.7)	5.2 (3.9–6.8)	4.1 (2.8–5.9)	3.7 (2.4–6.0)	4.4 (2.2–8.7)
Any anxiety disorder	1.9 (1.7–2.1)	1.1 (1.0–1.3)	2.8 (2.4–3.2)	1.7 (1.5–2.0)	1.1 (0.9–1.3)	2.6 (2.2–3.0)	2.8 (2.3–3.5)	1.7 (1.3–2.2)	6.2 (4.4–8.7)
Panic disorder									
With agoraphobia	3.1 (2.1–4.6)	1.9 (1.1–3.1)	4.2 (2.5–7.1)	2.5 (1.6–4.0)	1.4 (0.8–2.6)	3.6 (2.0–6.5)	6.0 (3.6–9.7)	3.5 (1.6–7.7)	10.5 (5.6–19.7)
Without agoraphobia	2.1 (1.6–2.6)	0.9 (0.8–1.3)	3.5 (2.6–4.7)	2.0 (1.5–2.6)	0.8 (0.5–1.2)	3.4 (2.5–4.7)	3.4 (2.4–5.0)	1.6 (0.9–3.0)	7.6 (4.7–12.2)
Social phobia	1.9 (1.5–2.4)	1.1 (0.8–1.5)	2.8 (2.1–3.7)	1.7 (1.3–2.1)	0.9 (0.7–1.3)	2.5 (1.8–3.3)	3.0 (2.2–4.1)	2.0 (1.3–3.0)	5.4 (3.5–8.3)
Specific phobia	1.6 (1.4–1.9)	1.1 (0.9–1.4)	2.2 (1.9–2.7)	1.6 (1.4–1.8)	1.1 (0.9–1.3)	2.2 (1.8–2.6)	2.3 (1.8–2.9)	1.6 (1.2–2.2)	3.8 (2.5–5.8)
Generalized anxiety disorder	2.3 (1.9–2.9)	1.1 (0.8–1.6)	3.8 (2.9–5.0)	1.9 (1.5–2.5)	0.9 (0.6–1.4)	3.1 (2.3–4.1)	4.6 (3.3–6.4)	2.1 (1.3–3.5)	10.4 (6.5–16.7)

* Data are given as odds ratios (ORs) (95% confidence interval). The ORs represent the odds of having a specific mood or anxiety disorder among individuals with a specific substance use disorder relative to the odds of having a specific mood or anxiety disorder among individuals who do not have the specific substance use disorder.

**Table 5:** Twelve-Month Prevalence of DSM–IV Independent Mood and Anxiety Disorders Among Respondents With a 12-Month DSM–IV Substance Use Disorder*

Comorbid Disorder	Index Disorder: Substance Use Disorder								

	Any Substance Use Disorder	Any Substance Abuse	Any Substance Dependence	Any Alcohol Use Disorder	Alcohol Abuse	Alcohol Dependence	Any Drug Use Disorder	Any Drug Abuse	Any Drug Dependence
Any mood disorder	19.67 (0.78)	12.33 (0.82)	29.19 (1.49)	18.85 (0.83)	11.73 (0.88)	27.55 (1.53)	31.80 (2.07)	21.23 (2.16)	55.02 (4.27)
Major depression	14.50 (0.68)	8.85 (0.71)	21.82 (1.40)	13.70 (0.73)	8.15 (0.74)	20.48 (1.43)	23.33 (1.84)	15.75 (1.91)	39.99 (3.95)
Dysthymia	3.54 (0.36)	2.08 (0.37)	5.43 (0.69)	2.93 (0.34)	1.54 (0.35)	4.63 (0.67)	8.37 (1.21)	4.59 (1.08)	16.68 (2.83)
Mania	4.94 (0.41)	2.39 (0.40)	8.25 (0.81)	4.66 (0.41)	2.23 (0.38)	7.63 (0.83)	9.99 (1.33)	6.34 (1.18)	18.00 (3.11)
Hypomania	3.30 (0.33)	2.04 (0.37)	4.94 (0.58)	3.30 (0.35)	1.92 (0.39)	4.99 (0.62)	4.30 (0.78)	4.07 (0.93)	4.81 (1.53)
Any anxiety disorder	17.71 (0.81)	12.45 (0.80)	24.54 (1.39)	17.05 (0.85)	11.81 (0.83)	23.45 (1.42)	25.36 (2.04)	17.33 (1.75)	43.02 (4.29)
Panic disorder									
With agoraphobia	1.46 (0.26)	1.00 (0.24)	2.05 (0.52)	1.25 (0.26)	0.77 (0.22)	1.84 (0.52)	2.98 (0.72)	1.90 (0.73)	5.35 (1.58)
Without agoraphobia	2.86 (0.29)	1.38 (0.27)	4.79 (0.60)	2.80 (0.31)	1.24 (0.27)	4.70 (0.63)	4.89 (0.82)	2.44 (0.71)	10.27 (2.16)
Social phobia	4.72 (0.46)	3.09 (0.45)	6.83 (0.87)	4.25 (0.46)	2.61 (0.42)	6.25 (0.85)	7.59 (1.08)	5.17 (1.01)	12.91 (2.43)
Specific phobia	10.54 (0.67)	7.82 (0.71)	14.06 (1.12)	10.40 (0.69)	7.58 (0.72)	13.84 (1.15)	14.55 (1.57)	11.05 (1.50)	22.26 (3.78)
Generalized anxiety disorder	4.20 (0.41)	2.24 (0.39)	6.74 (0.80)	3.60 (0.40)	1.90 (0.39)	5.69 (0.71)	8.28 (1.25)	4.21 (1.00)	17.22 (3.35)

* Data are given as a percentage of respondents (SE).

**Table 6:** Twelve-Month Prevalence of DSM–IV Substance Use Disorders Among Respondents With a 12-Month DSM–IV Independent Mood or Anxiety Disorder*

Comorbid Disorder	Index Disorder: Mood or Anxiety Disorder										

	Any Mood Disorder	Major Depression	Dysthymia	Mania	Hypomania	Any Anxiety Disorder	Panic Disorder With Agoraphobia	Panic Disorder Without Agoraphobia	Social Phobia	Specific Phobia	Generalized Anxiety Disorder
Any substance use disorder	19.97 (0.78)	19.20 (0.85)	18.07 (1.66)	27.91 (2.13)	26.62 (2.33)	14.96 (0.66)	24.15 (3.62)	17.30 (1.69)	16.05 (1.44)	13.83 (0.81)	19.08 (1.68)
Any substance abuse	7.05 (0.46)	6.61 (0.52)	6.00 (1.04)	7.61 (1.27)	9.29 (1.68)	5.93 (0.39)	9.32 (2.18)	4.69 (0.92)	5.93 (0.84)	5.79 (0.53)	5.74 (0.95)
Any substance dependence	12.91 (0.70)	12.59 (0.83)	12.07 (1.40)	20.30 (1.89)	17.33 (1.85)	9.02 (0.55)	14.83 (3.33)	12.60 (1.59)	10.12 (1.25)	8.04 (0.64)	13.34 (1.51)
Any alcohol use disorder	17.30 (0.75)	16.40 (0.82)	13.54 (1.51)	23.79 (1.94)	24.04 (2.27)	13.02 (0.65)	18.81 (3.42)	15.29 (1.62)	13.05 (1.30)	12.34 (0.79)	14.82 (1.54)
Alcohol abuse	5.92 (0.43)	5.37 (0.47)	3.92 (0.87)	6.26 (1.07)	7.68 (1.58)	4.96 (0.37)	6.38 (1.84)	3.73 (0.82)	4.41 (0.69)	4.95 (0.49)	4.30 (0.85)
Alcohol dependence	11.38 (0.67)	11.03 (0.80)	9.62 (1.31)	17.52 (1.81)	16.36 (1.84)	8.06 (0.56)	12.42 (3.19)	11.56 (1.55)	8.64 (1.16)	7.39 (0.62)	10.52 (1.30)
Any drug use disorder	6.90 (0.56)	6.61 (0.63)	9.14 (1.32)	12.06 (1.60)	7.42 (1.35)	4.58 (0.41)	10.58 (2.36)	6.32 (1.08)	5.52 (0.76)	4.08 (0.47)	8.06 (1.22)
Any drug abuse	3.17 (0.56)	3.07 (0.40)	3.45 (0.83)	5.26 (0.96)	4.82 (1.11)	2.15 (0.23)	4.65 (1.75)	2.17 (0.65)	2.59 (0.49)	2.13 (0.30)	2.82 (0.65)
Any drug dependence	3.74 (0.40)	3.54 (0.44)	5.70 (0.99)	6.80 (1.25)	2.59 (0.83)	2.43 (0.33)	5.94 (1.71)	4.16 (0.88)	2.94 (0.57)	1.95 (0.39)	5.24 (1.14)

* Data are given as a percentage of respondents (SE).

**Table 7:** Twelve-Month Prevalence of DSM–IV Substance Use Disorders Among Respondents With 12-Month DSM–IV Independent Mood and Anxiety Disorders Who Sought Treatment in the Past 12 Months

Disorder	Respondents, % (S.E.)
Those With Any Mood Disorder (25.81%)*	
Any substance use disorder	20.78 (1.55)
Any alcohol use disorder	17.48 (1.49)
Any drug use disorder	7.96 (1.14)
Those With Major Depression (28.46%)*	
Any substance use disorder	20.34 (1.67)
Any alcohol use disorder	16.80 (1.57)
Any drug use disorder	7.54 (1.18)
Those With Those With Dysthymia (33.20%)*	
Any substance use disorder	18.54 (3.08)
Any alcohol use disorder	14.78 (2.91)
Any drug use disorder	6.20 (1.59)
Those With Mania (21.91%)*	
Any substance use disorder	22.47 (3.87)
Any alcohol use disorder	18.89 (3.67)
Any drug use disorder	10.34 (2.87)
Those With Hypomania (3.78%)*	
Any substance use disorder	30.97 (12.44)
Any alcohol use disorder	30.97 (12.44)
Any drug use disorder	0.00 (0.00)
Those With Any Anxiety Disorder (12.13%)*	
Any substance use disorder	16.51 (1.95)
Any alcohol use disorder	12.49 (1.83)
Any drug use disorder	7.26 (1.27)
Those With Panic Disorder With Agoraphobia (39.19%)*	
Any substance use disorder	21.89 (5.02)
Any alcohol use disorder	15.39 (4.39)
Any drug use disorder	9.67 (3.00)
Those With Panic Disorder Without Agoraphobia (29.97%)*	
Any substance use disorder	15.38 (2.82)
Any alcohol use disorder	13.71 (2.69)
Any drug use disorder	5.14 (1.66)
Those With Social Phobia (11.33%)*	
Any substance use disorder	21.32 (4.86)
Any alcohol use disorder	15.97 (4.63)
Any drug use disorder	8.15 (2.44)
Those With Specific Phobia (3.44%)*	
Any substance use disorder	16.03 (3.76)
Any alcohol use disorder	11.47 (3.40)
Any drug use disorder	6.12 (2.46)
Those With Generalized Anxiety Disorder (27.15%)*	
Any substance use disorder	15.92 (2.78)
Any alcohol use disorder	10.10 (2.60)
Any drug use disorder	9.70 (2.40)

* Data in parentheses are the percentages of respondents with the index disorders who sought treatment in the past 12 months.

**Table 8:** Twelve-Month Prevalence of DSM–IV Independent Mood and Anxiety Disorders Among Respondents With DSM–IV Substance Use Disorders Who Sought Treatment in the Past 12 Months

Disorder	Respondents, % (S.E.)
Those With Any Alcohol Use Disorder (5.81%)*	
Any mood disorder	40.69 (4.11)
Major depression	32.75 (4.01)
Dysthymia	11.01 (2.74)
Mania	12.56 (2.81)
Hypomania	3.07 (1.37)
Any anxiety disorder	33.38 (4.17)
Panic disorder	
With agoraphobia	4.10 (1.54)
Without agoraphobia	9.10 (2.48)
Social phobia	8.49 (2.54)
Specific phobia	17.24 (3.10)
Generalized anxiety disorder	12.35 (3.01)
Any drug use disorder	33.05 (4.23)
Those With Any Drug Use Disorder (13.10%)*	
Any mood disorder	60.31 (5.86)
Major depression	44.26 (6.28)
Dysthymia	25.91 (5.19)
Mania	20.39 (5.17)
Hypomania	2.48 (1.67)
Any anxiety disorder	42.63 (5.97)
Panic disorder	
With agoraphobia	5.92 (2.19)
Without agoraphobia	8.64 (3.05)
Social phobia	12.09 (3.48)
Specific phobia	22.52 (4.99)
Generalized anxiety disorder	22.07 (5.18)
Any alcohol use disorder	55.16 (6.29)

* Data in parentheses are the percentages of respondents with the substance use disorders who sought treatment in the past 12 months.

## References

[1] Grant BF (1995). Comorbidity between DSM–IV drug use disorders and major depression: results of a national survey of adults. J Subst Abuse.

[2] Kessler RC, Nelson CB, McGonagle KA, Edlund MJ, Frank RG, Leaf PJ (1996). The epidemiology of co-occurring addictive and mental disorders. Am J Orthopsychiatry.

[3] Regier DA, Farmer ME, Rae DS, Locke BZ, Keith SJ, Judd LL, Goodwin FK (1990). Comorbidity of mental disorders with alcohol and other drug abuse: results from the Epidemiologic Catchment Area (ECA) Study. JAMA.

[4] Goetzel RZ, Hawkins K, Ozminkowski RJ (2003). The health and productivity cost burden of the “top 10” physical and mental conditions affecting six large US employers in 1999. J Occup Environ Med.

[5] Roy-Byrne PP, Stang P, Wittchen HU, Ustun BT, Walters EE, Kessler RC (2000). Lifetime panic-depression comorbidity in the National Comorbidity Survey: association with symptoms, impairment, course and help seeking. Br J Psychiatry.

[6] Sanderson K, Andrews G (2002). Prevalence and severity of mental health disability and relationship to diagnosis. Psychiatr Serv.

[7] Stewart WF, Ricci JA, Chee E, Hahn SR, Morganstein D (2003). Cost of lost productive work time among US workers with depression. JAMA.

[8] Hirschfield R, Hasin D, Keller M, Endicott J, Wunder J, Maser J, Cloninger C (1990). Depression and alcoholism: comorbidity in a longitudinal study. Comorbidity of Mood and Anxiety Disorders.

[9] Merikangas K, Stevens DE, Wetherington CL, Roman AB (1998). Substance abuse among women: familial factors and comorbidity. Drug Addiction Research and the Health of Women.

[10] Svanum S, McAdoo WG (1989). Predicting rapid relapse following treatment for chemical dependence: a matched-subjects design. J Consult Clin Psychol.

[11] Swendson JD, Merikangas KR (2000). The comorbidity of depression and substance use disorders. Clin Psychol Rev.

[12] Hasin DS, Nunes E, Kranzler HR, Rounsaville BJ (1997). Comorbidity of alcohol, drug and psychiatric disorders: epidemiology. Dual Diagnosis and Treatment Substance Abuse and Comorbid Mental and Psychiatric Disorders.

[13] Hasin DS, Grant BF (2002). Major depression in 6,050 former drinkers. Arch Gen Psychiatry.

[14] Kadden RM, Kranzler HR, Rounsaville BS (1995). Validity of the distinction between substance-induced and independent depression and anxiety disorders. Am J Addict.

[15] Raimo EB, Schuckit MA (1998). Alcohol dependence and mood disorders. Addict Behav.

[16] Schuckit MA, Tipp JE, Bergman M, Reich W, Hesselbrock VM, Smith TL (1997). Comparison of induced and independent major depressive disorder in 2,945 alcoholics. Am J Psychiatry.

[17] Schuckit MA, Tipp JE, Bucholz KK, Nurnberger JI, Hesselbrock VM, Crowe RR, Kramer J (1997). The life-time rates of three major mood disorders and four major anxiety disorders in alcoholics and controls. Addiction.

[18] American Psychiatric Association (1994). Diagnostic and Statistical Manual of Mental Disorders.

[19] Robins LN, Regier DS (1991). Psychiatric Disorders in America: The Epidemiologic Catchment Area Study.

[20] American Psychiatric Association (1980). Diagnostic and Statistical Manual of Mental Disorders.

[21] American Psychiatric Association (1987). Diagnostic and Statistical Manual of Mental Disorders.

[22] Kessler RC, Walters EE, Tsuang MT, Tohen M (2002). The National Comorbidity Survey. Textbook in Psychiatric Epidemiology.

[23] Helzer J, Pryzbeck TR (1988). The co-occurrence of alcoholism with other psychiatric disorders in the general population and its impact on treatment. J Stud Alcohol.

[24] Grant BF, Harford TC, Dawson DA, Chou SP, Dufour M, Pickering RP (1992). Prevalence of DSM–IV alcohol abuse and dependence: United States, 1992. Alcohol Health Res World.

[25] Grant BF, Moore TC, Kaplan K (2003). Source and Accuracy Statement: Wave 1 National Epidemiologic Survey on Alcohol and Related Conditions (NESARC).

[26] Grant BF, Stinson FS, Dawson DA, Chou SP, Ruan WJ, Pickering RP (2005). Co-occurrence of 12-month alcohol and drug use disorders and personality disorders in the United States: results from the National Epidemiologic Survey on Alcohol and Related Conditions. Arch Gen Psychiatry.

[27] Grant BF, Dawson DA, Hasin DS (2001). The Alcohol Use Disorder and Associated Disabilities Interview Schedule—DSM–IV Version.

[28] Canino GJ, Bravo M, Ramfrez R, Febo V, Fernandez R, Hasin D (1999). The Spanish Alcohol Use Disorder and Associated Disabilities Interview Schedule (AUDADIS): reliability and concordance with clinical diagnoses in a Hispanic population. J Stud Alcohol.

[29] Chatterji S, Saunders JB, Vrasti R, Grant BF, Hasin DS, Mager D (1997). The reliability of the Alcohol Use Disorders and Associated Disabilities Interview Schedule–Alcohol/Drug–Revised (AUDADIS–ADR) in India, Romania and Australia. Drug Alcohol Depend.

[30] Grant BF, Dawson DA, Stinson FS, Chou PS, Kay W, Pickering R (2003). The Alcohol Use Disorder and Associated Disabilities Interview, Schedule–IV (AUDADIS–IV): reliability of alcohol consumption, tobacco use, family history of depression and psychiatric diagnostic modules in a general population sample. Drug Alcohol Depend.

[31] Grant BF, Harford TC, Dawson DA, Chou PS, Pickering R (1995). The Alcohol Use Disorder and Associated Disabilities Interview Schedule (AUDADIS): reliability of alcohol and drug modules in a general population sample. Drug Alcohol Depend.

[32] Hasin D, Carpenter KM, McCloud S, Smith M, Grant BF (1997). The Alcohol Use Disorder and Associated Disabilities Interview Schedule (AUDADIS): reliability of alcohol and drug modules in a clinical sample. Drug Alcohol Depend.

[33] Grant BF (1992). DSM–III–R and proposed DSM–IV alcohol abuse and dependence, United States, 1992: a nosological comparison. Alcohol Clin Exp Res.

[34] Grant BF (1993). DSM–III–R and proposed DSM–IV harmful use of alcohol/alcohol abuse and dependence, United States 1988: a nosological comparison. Alcohol Clin Exp Res.

[35] Grant BF (1996). DSM–IV, DSM–III and ICD–10 alcohol and drug abuse, harmful use and dependence, United States, 1992: a nosological comparison. Alcohol Clin Exp Res.

[36] Grant BF (1993). The relationship between ethanol intake and DSM–III–R alcohol dependence: results of a national survey. J Subst Abuse.

[37] Grant BF, Harford TC (1990). The relationship between ethanol intake and DSM–III–R alcohol dependence. J Stud Alcohol.

[38] Grant BF, Harford TC (1988–89). The relationship between ethanol intake and DSM–III alcohol use disorders: a cross-perspective analysis. J Subst Abuse.

[39] Hasin DS, Grant BF (1994). Draft criteria for alcohol use disorders: comparison to DSM–III–R and implications. Alcohol Clin Esp Res.

[40] Hasin DS, Grant BF (1994). Nosological comparisons of DSM–III–R and DSM–IV alcohol abuse and dependence in a clinical facility: comparison to National HIS88 results. Alcohol Clin Exp Res.

[41] Hasin D, Grant BF, Cottler L, Blaine J, Towle L, Ustun B, Sartorius N (1997). Nosological comparisons of alcohol and drug diagnoses: a multisite, multi-instrument international study. Drug Alcohol Depend.

[42] Hasin D, Li Q, McCloud S, Endicott J (1996). Agreement between DSM–III, DSM–III–R, DSM–IV and ICD–l0 alcohol diagnoses in a US community sample of heavy drinkers. Addiction.

[43] Hasin DS, Van Rossem R, McCloud S, Endicott J (1997). Alcohol dependence and abuse diagnoses: validity in a community sample of heavy drinkers. Alcohol Clin Exp Res.

[44] Cottler LB, Grant BF, Blaine J, Mavreas V, Pull CB, Hasin D, Compton WM, Rubio-Stipee M, Mager D (1997). Concordance of DSM-IV alcohol and drug use disorder criteria and diagnoses as measured by AUDADIS–ADR, CIDI and SCAN. Drug Alcohol Depend.

[45] Hasin DS, Paykin A (1999). Alcohol dependence and abuse diagnoses: concurrent validity in a nationally representative sample. Alcohol Clin Res.

[46] Pull CB, Saunders JB, Mavreas V, Cottler LB, Grant BF, Hasin DS, Blaine J, Mager D, Ustun BT (1997). Concordance between ICD–10 alcohol and drug use disorder criteria and diagnoses as measured by the AUDADIS–ADR, CIDI and SCAN: results of a cross-national study. Drug Alcohol Depend.

[47] Hasin DS, Muthen B, Grant BF, Vrasti R (1997). The dimensionality of DSM–IV alcohol abuse and dependence: factor analysis in a clinical sample. Alcoholism: New Research Perspectives.

[48] Muthen BO, Grant B, Hasin D (1993). The dimensionality of alcohol abuse and dependence: factor analysis of DSM–III–R and proposed DSM–IV criteria in the 1988 National Health Interview Survey. Addiction.

[49] Nelson CB, Rehm J, Ustun B, Grant BF, Chatterji S (1999). Factor structure for DSM–IV substance disorder criteria endorsed by alcohol, cannabis, cocaine and opiate users: results from the World Health Organization Reliability and Validity Study. Addiction.

[50] Harford TC, Grant BF (1994). Prevalence and population validity of DSM–III–R alcohol abuse and dependence: the 1989 National Longitudinal Survey on Youth. J Subst Abuse.

[51] Vrasti R, Grant BF, Chatterji S, Ustun BT, Mager D, Olteanu I, Badoi M (1998). Reliability of the Romanian version of the alcohol module of the WHO Alcohol Use Disorder and Associated Disabilities Interview Schedule–Alcohol/Drug–Revised. Eur Addict Res.

[52] Ustun B, Compton W, Mager D, Babor T, Balyewu O, Chatterji S, Cottler L, Gogus A, Mavreas V, Peters L, Pull C, Saunders J, Smeets R, Stipee MR, Vrasti R, Hasin D, Room R, Van den Brink W, Regier D, Blaine J, Grant BF, Sartorius N (1997). WHO study on the reliability and validity of the alcohol and drug use disorder instruments: overview of methods and results. Drug Alcohol Depend.

[53] Hasin DS, Schuckit MA, Martin CS, Grant BF, Bucholz KK, Helzer JE (2003). The validity of DSM–IV alcohol dependence: what do we know and what do we need to know?. Alcohol Clin Exp Res.

[54] Ware JE, Kosinski M, Turner Bowker DM, Gandek B (2002). How to Score Version 2 of the SF-12 Health Survey.

[55] Research Triangle Institute (2002). Software for Survey Data Analysis (SUDAAN), Version 8.0.

[56] Hasin DS, Liu X-H, Nunes E, McCloud S, Samet S, Endicott J (2002). Effects of major depression on remission and relapse of substance dependence. Arch Gen Psychiatry.

[57] Aharonovich E, Liu X, Nunes E, Hasin D (2002). Suicide attempts in substance abusers: effects of major depression in relation to substance use disorders. Am J Psychiatry.

[58] Cornelius JR, Salloum IM, Haskett RF, Daley DC, Cornelius MD, Thase ME, Perel JM (2000). Fluoxetine versus placebo in depressed alcoholics: a 1-year follow-up study. Addict Behav.

[59] Kranzler HR, Burleson JA, Korner P, Del Boca FK, Bohn MJ, Brown J, Liebowitz N (1995). Placebo-controlled trial of fluoxetine as an adjunct to relapse prevention in alcoholics. Am J Psychiatry.

[60] Mason BJ, Kocsis JH, Ritvo EC, Cutter RB (1996). A double-blind, placebo-controlled trial of desipramine for primary alcohol dependence stratified on presence or absence of major depression. JAMA.

[61] McGrath PJ, Nunes EV, Stewart JW, Goldman D, Agosti V, Ocepek-Welikson K, Quitkin FM (1996). Imipramine treatment of alcoholics with primary depression: a placebo-controlled clinical trial. Arch Gen Psychiatry.

[62] Nunes EV, Quitkin FM, Donovan SJ, Deliyannides D, Ocepek-Welikson K, Koenig T, Brady R, McGrath PJ, Woody G (1998). Imipramine treatment of opiate-dependent patients with depressive disorders: a placebo-controlled trial. Arch Gen Psychiatry.

